# Weathering the hunt: The role of barometric pressure in predator insects' foraging behaviour

**DOI:** 10.1002/ece3.10416

**Published:** 2023-08-09

**Authors:** Kamila E. X. Azevedo, Diego M. Magalhães, Rafael de Andrade Moral, José Maurício S. Bento

**Affiliations:** ^1^ Department of Entomology and Acarology, “Luiz de Queiroz” College of Agriculture University of São Paulo Piracicaba Brazil; ^2^ Department of Mathematics and Statistics Maynooth University Maynooth Ireland

**Keywords:** abiotic factors, biological control, *Chrysoperla externa*, *Doru luteipes*, *Eriopis connexa*, life stages

## Abstract

Abiotic factors strongly influence ecological interactions and the spatial distribution of organisms. Despite the essential role of barometric pressure, its influence on insect behaviour remains poorly understood, particularly in predators. The effect of barometric pressure variation can significantly impact biological control programs involving entomophagous insects, as they must efficiently allocate time and energy to search for prey in challenging environments. We investigated how predatory insects from different taxonomic groups (Coleoptera, Dermaptera and Neuroptera) adapt their foraging behaviour in response to variations in barometric pressure (low, medium and high). We also examined the response of different life stages to changes in pressure regimes during foraging activities. Our results showed that the searching time of *Doru luteipes* (Dermaptera: Forficulidae) was faster in a favourable high‐pressure regime, whereas *Chrysoperla externa* (Neuroptera: Chrysopidae) and *Eriopis connexa* (Coleoptera: Coccinellidae) had similar searching times under varying pressure regimes. Although no differences in prey feeding time were observed among the studied species, the consumption rate was influenced by low barometric pressure leading to a decrease in the number of preyed eggs. Moreover, we provide novel insights into how hemimetabolous (*D. luteipes*) and holometabolous (*E. connexa*) species at different life stages respond to barometric pressure. *Doru luteipes* nymphs and adults had similar consumption rates across all pressure regimes tested, whereas *E. connexa* larvae consumed fewer eggs under low barometric pressure, but adults were unaffected. This highlights the importance of investigating how abiotic factors affect insects foraging efficiency and predator–prey interactions. Such studies are especially relevant in the current context of climate change, as even subtle changes in abiotic factors can have strong effects on insect behaviour. Barometric pressure is a key meteorological variable that serve as a warning signal for insects to seek shelter and avoid exposure to weather events that could potentially increase their mortality. Understanding the effects of barometric pressure on predatory insects' behaviour can help us develop more effective pest management strategies and promote the resilience of agroecosystems. We provide new insights into the complex relationship between barometric pressure and predator–prey interactions.

## Graphical abstract

Despite the essential role of barometric pressure, its influence on insects remains poorly understood, particularly in predator insects. Our study showed that three predator species from distinct orders, with varying morphological, physiological, ecological and biological characteristics, modify their foraging behaviour in response to barometric pressure conditions. Understanding the effects of barometric pressure on predator insects' behaviour can help us develop more effective pest management strategies and promote the resilience of agroecosystems in the face of climate change.

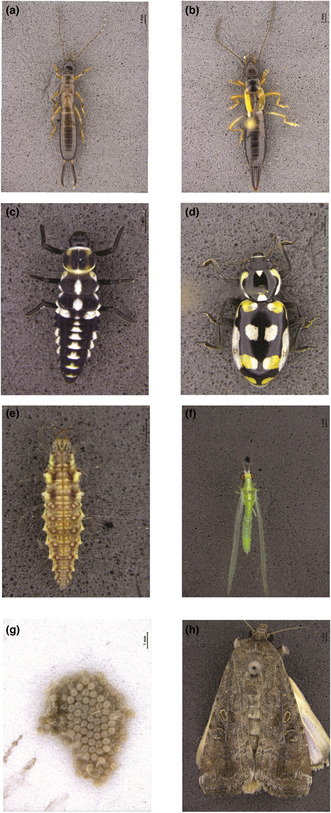

## 1 INTRODUCTION

Animals are continually exposed to various biotic and abiotic stresses throughout their lives, which can have profound effects on their survival, physiology, morphology, biology and behaviour (Wingfield, 2013). Abiotic factors such as temperature, humidity, solar radiation, wind and precipitation have been shown to strongly influence ecological interactions and the spatial distribution of organisms (Price et al., 2011). Atmospheric pressure, which is the force exerted by the weight of the atmosphere on the Earth's surface (Lazaridis, 2011), is highly correlated with temperature variation, wind and precipitation (Barry & Chorley, 2009). High atmospheric pressure is associated with stable and dry weather, while low‐pressure values represent less stability in the atmosphere, which can lead to the formation of clouds and increased chances of turbulent weather, such as stronger winds, precipitation and storms (Lutgens & Tarbuck, 2012; Wellington, 1946). Thus, changes in atmospheric pressure can have a significant impact on meteorological conditions, which in turn can strongly affect animal behaviour (Ariano‐Sánchez et al., 2022; Breuner et al., 2013; Heupel et al., 2003; Pellegrino et al., 2013).

Animals, including insects, respond to changes in atmospheric pressure by modulating their behaviour (Crespo & Castelo, 2012). Increasing atmospheric pressure can enhance mating (Pellegrino et al., 2013), learning (Dagaeff et al., 2016), flight (Fournier et al., 2005) and feeding behaviours (Costa et al., 2022). Conversely, a decrease in atmospheric pressure can lead to a reduction in activity due to unsettled weather and increased mortality risk (Jones et al., 2018). Research has shown that various insect orders, such as Diptera, Orthoptera, Coleoptera, Thysanoptera, Hemiptera and Hymenoptera, exhibit changes in behaviour due to atmospheric pressure variations (Austin et al., 2014; Costa et al., 2022; Dagaeff et al., 2016; Fournier et al., 2005; Jones et al., 2018; Lanier & Burns, 1978; McFarlane et al., 2015; Musiolek & Kočárek, 2016; Zagvazdina et al., 2015). For example, the Asian citrus psyllid (*Diaphorina citri* Kuwayama) can increase its dispersal capacity when atmospheric pressure increases by 4.57 mbar/h but decrease it when the pressure drops by 5.47 mbar/h (Martini & Stelinski, 2017). The cucurbit beetle, *Diabrotica speciosa* (Germar), exhibits reduced locomotory activity under low atmospheric pressure, while the true armyworm, *Pseudaletia unipuncta* (Haworth), and the potato aphid, *Macrosiphum euphorbiae* (Thomas), decrease their calling behaviour (Pellegrino et al., 2013). However, there has been relatively little research on how atmospheric pressure affects the behaviour of natural enemies. For parasitoid wasps, it has been reported that atmospheric pressure can affect the flight capacity of two Trichogrammatidae species (Fournier et al., 2005), the mate‐seeking behaviour of *Aphidius nigripes* Ashmead (Marchand & McNeil, 2000), the foraging of *Mallophora ruficauda* (Wiedemann) (Crespo & Castelo, 2012), *Cotesia glomerata* L. (Steinberg et al., 1992; Vosteen et al., 2020), and *Fopius arisanus* (Sonan) (Rousse et al., 2009), and host discrimination of *Leptopilina heterotma* (Thomson) (Roitberg et al., 1993). The effect of atmospheric pressure on predatory insects, however, has not been reported. For other predators, such as bats, birds and sharks, changes in atmospheric pressure can directly affect many aspects of their behaviour and physiology (Bender & Hartman, 2015; Breuner et al., 2013; Heupel et al., 2003).

Entomophagous insects play a vital role in maintaining the functioning and structure of ecosystems by controlling herbivorous insects (Parra et al., 2002). However, during foraging, predator insects face various risks, including both biotic and abiotic factors. To mitigate these risks, they may adjust their foraging behaviour by altering their prey selection, foraging time and location, and consumption rate, which ultimately affects their predatory efficiency (Ferran & Dixon, 2013; King & Marshall, 2022). The increased adoption of sustainable agricultural practices has led to a rise in research on the foraging behaviour of entomophagous insects (Perennes et al., 2023). Yet, there is still a need to understand how various biotic and abiotic factors, including atmospheric pressure, affect their foraging behaviour to better comprehend the dynamics of predator–prey interactions and ecosystem functioning. Atmospheric pressure is a powerful predictor that reflects weather conditions and can have a significant impact on predator behaviour (Skendžić et al., 2021). Moreover, considering the different responses to atmospheric pressure variations among life stages of predatory insects is crucial for understanding the ecological implications of environmental changes, as these responses can potentially affect predator–prey interactions, population dynamics and overall ecosystem functioning. In the current context of climate change, such studies are of great importance, as even subtle changes in abiotic factors can have strong effects on insects (Vosteen et al., 2020). Therefore, variations in atmospheric pressure can significantly impact biological control programs involving entomophagous insects, as these insects face environmental risk factors to efficiently allocate their time and energy towards the exploration, search and location of their prey.

Here, we investigated the foraging behaviour of three predatory insects from different taxonomic groups, namely the earwig *Doru luteipes* (Scudder) (Dermaptera: Forficulidae), the ladybird beetle *Eriopis connexa* (Germar) (Coleoptera: Coccinelidae) and the green lacewing *Chrysoperla externa* Hagen (Neuroptera: Chrysopidae), in response to varying regimes of barometric pressure (i.e. a measurement of atmospheric pressure). These species were carefully selected based on their unique morphological, behavioural, developmental and dietary characteristics. Our research aimed to answer the following questions:

1. How does the foraging behaviour of predatory insects change between different life stages (immature and adult) in response to varying barometric pressure regimes (low, medium and high)?
2. Is there a significant difference in the consumption rate of predatory insects across different barometric pressure regimes (low, medium and high)?

## 2 MATERIALS AND METHODS

### 2.1 Study site

The experiments were conducted in Piracicaba, SP, Brazil, situated at an altitude of 546 m above sea level. The city is located at a latitude of 22°42′30″ S and longitude of 47°38′00″ W. According to the Köppen classification, it falls under the tropical category with a dry winter (Aw) (Alvares et al., 2022). The region receives an average annual precipitation of 1382 mm, which can occur throughout the year, with the highest volumes observed during the summer months (December–March). The average barometric pressure is 950 ± 1 mbar (www.leb.esalq.usp.br/leb/base.html). Fluctuations in barometric pressure of −8 mbar reflect atmospheric instability, typically accompanied by the formation of clouds, stronger winds and intense rainfall. Conversely, an increase of +8 mbar indicates stable weather conditions (Costa et al., 2022).

### 2.2 Predators rearing

Laboratory colonies of *D. luteipes* were established from field collections in commercial maize farms located in Piracicaba, SP, Brazil. Adults and larvae were kept in plastic containers (10 × 24 × 35 cm) with an organza fabric lid to ensure proper ventilation, and brown paper to reduce the amount of light. The earwigs were fed ad libitum with an artificial diet based on cat food (35%), wheat bran (27%), brewer's yeast (23%), powdered milk (14%), Nipagin (0.5%) and ascorbic acid (0.5%) (Guimarães, 2006). Cotton plugs soaked in water were provided as moisture. Refuges made of accordion‐shaped paper and small cardboard boxes (5 × 20 cm) were placed inside the rearing containers to prevent cannibalism and to accommodate the earwig's thigmotropic habit. Pieces of straw filled with moistened cotton were provided as oviposition substrates (Naranjo‐Guevara et al., 2017). The rearing room was maintained at 25 ± 1°C, 60 ± 10% RH, and a L:D 12:12 photoperiod.

Adults of *C. externa* were obtained from the Laboratory of Biological Control with Entomophages, Federal University of Lavras, in Lavras, MG, Brazil. Predators were kept in PVC (polyvinyl chloride) containers (20 × 15 cm), which were lined with white bond paper for oviposition and sealed with a voile fabric lid (Amaral et al., 2013). An artificial diet of beer yeast and honey (1:1 v/v) and moistened cotton were provided as food and moisture, respectively. Green lacewing larvae were individualized in glass tubes (8.5 × 2.5 cm) and fed ad libitum with eggs of *Anagasta kuehniella* (Zeller) (Lepidoptera: Pyralidae). Insects were maintained under controlled conditions (25 ± 1°C, 60 ± 10% RH, and a L:D 12:12 photoperiod).

Adults of *E. connexa* were obtained from the Laboratory of Insect Biology, University of São Paulo, in Piracicaba, SP, Brazil. Insects were kept in plastic containers (1000 mL) lined with bond paper and sealed with a voile fabric lid. Ladybird beetle adults were fed ad libitum with eggs of *A. kuehniella* and an artificial diet of beer yeast and honey (1:1 v/v) (Matos et al., 2022). To prevent cannibalism, larvae were individualized in glass tubes (8.5 × 2.5 cm) and fed ad libitum with eggs of *A. kuehniella*. Insects were maintained under controlled conditions (25 ± 1°C, 60 ± 10% RH, and a L:D 12:12 photoperiod).

### 2.3 Prey rearing

**FIGURE 1:**
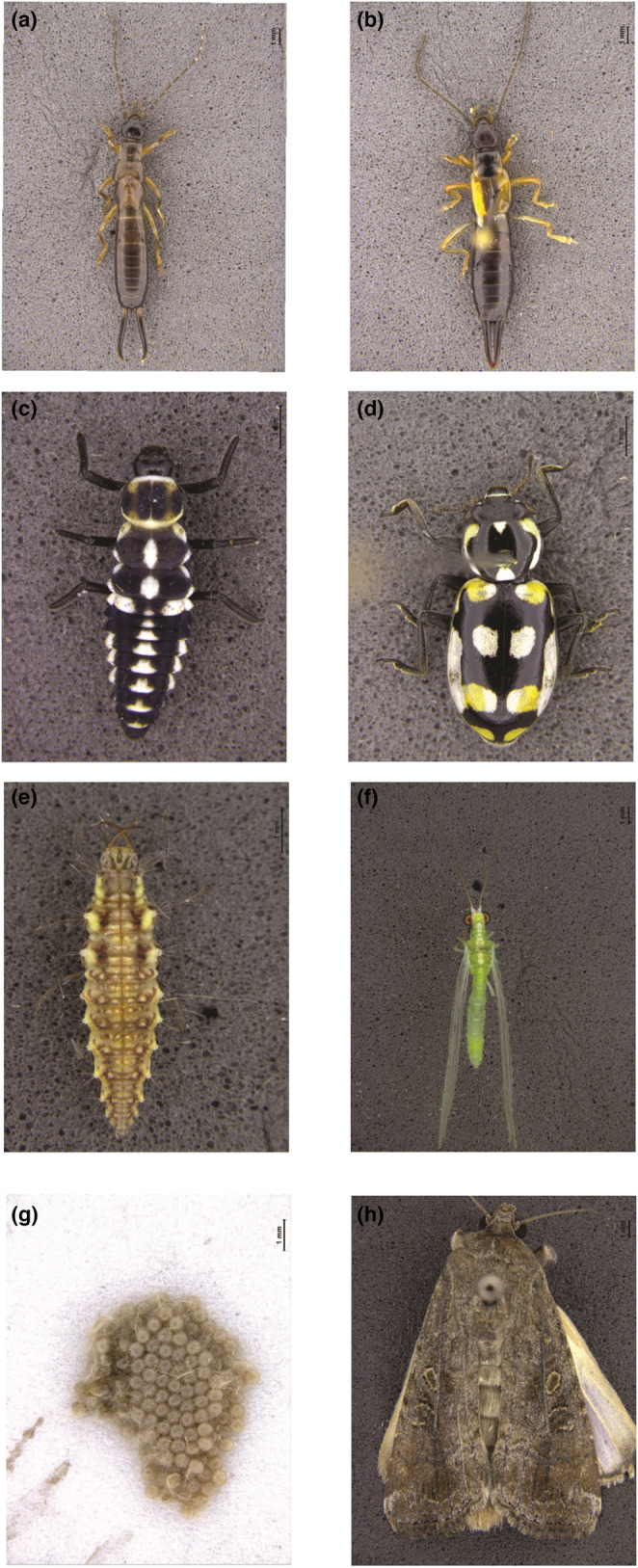
Predatory insects and prey used in the experiments to assess the effect of barometric pressure on their foraging behaviour. (a) Fourth instar nymph of *Doru luteipes*; (b) Female *Doru luteipes*; (c) Fourth instar larva of *Eriopis connexa*; (d) Female *Eriopis connexa*; (e) Third instar larva of *Chrysoperla externa*; (f) Female *Chrysoperla externa*; (g) *Spodoptera frugiperda* eggs; and (h). Female *Spodoptera frugiperda*.

*Spodotpera frugiperda* (J.E. Smith) (Lepidoptera: Noctuidae) is a common prey for *D. luteipes*, *C. externa* and *E. connexa* (Pacheco et al., 2021; Silva et al., 2013; Tavares et al., 2011) (Figure 1). Adults were obtained from the Laboratory of Insect Biology, University of São Paulo, in Piracicaba, SP, Brazil, and kept in PVC containers (20 × 15 cm) lined with bond paper and sealed with a voile fabric lid. An aqueous honey solution (10%) was provided as a food source for adults on a small ball of cotton wool inside the rearing container. First and second instar larvae were kept in a different plastic container (500 mL) with an artificial diet (Greene et al., 1976). Larvae from the other instars were individualized in a 50‐mL plastic container with an artificial diet until they pupate. The rearing room was maintained at 25 ± 1°C, 60 ± 10% RH, and a L:D 12:12 photoperiod.

### 2.4 Measurement of barometric pressure

**FIGURE 2:**
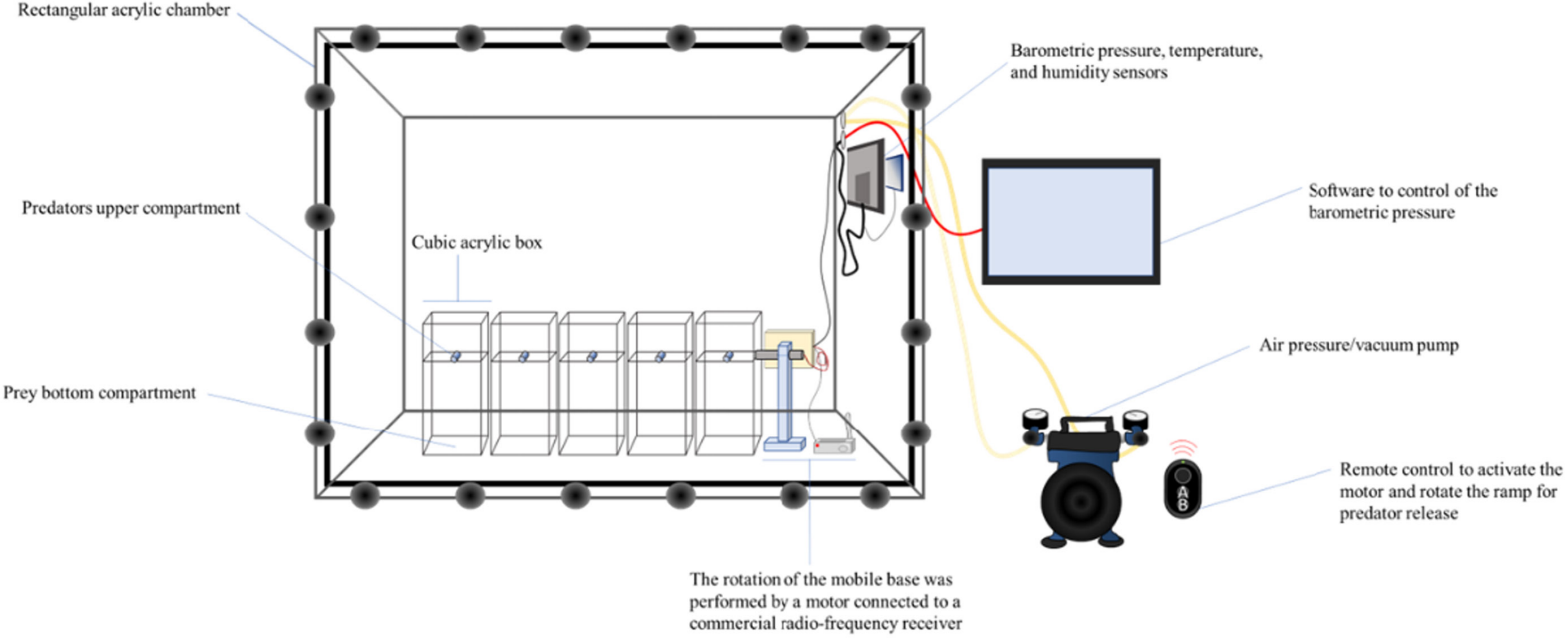
Schematic of the barometric pressure chamber and automated release device to test the influence of pressure on the foraging activity of *Doru luteipes*, *Eriopis connexa* and *Chrysoperla externa*.

The average and maximum annual variation of barometric pressure in Piracicaba, SP, Brazil, were determined using data from the meteorological station ‘Professor Jesus Marden dos Santos’ at the University of São Paulo. The experiments were conducted in an acrylic barometric chamber (40 × 90 × 70 cm) equipped with an automated pressure control system, as designed by Costa et al. (2022) (Figure 2). A vacuum‐pressure pump was utilized to regulate the pressure within the chamber. Three distinct pressure regimes were employed in the experiments. The medium‐pressure regime was set at the region's average barometric pressure of 950 mbar. For the low‐pressure regime (942 mbar), the pressure was reduced by −8 mbar, while for the high‐pressure regime (958 mbar), it was increased by +8 mbar. These adjustments were based on natural variations observed in the region (www.leb.esalq.usp.br/leb/base.html).

### 2.5 Impact of barometric pressure on predator foraging behaviour

To evaluate the influence of barometric pressure on predators' foraging behaviour, we used 4th instar immatures and 24‐h‐old females, except for *C. externa*, as adults do not exhibit a predatory habit (Dantas et al., 2021). Hence, 3rd instar larvae were used for *C. externa* (Figure 1). All predators were starved for 24 h before the assays. Within the barometric chamber (Figure 2), five automated release devices (27 × 11 × 11 cm) were installed. Each device consisted of two compartments separated by a revolving ramp, which could be activated remotely. The predators were placed in the upper compartment, while the prey were located in the bottom compartment on a filter paper disc (9 cm diameter). When released, predators would descend vertically into the bottom compartment and gain access to the prey. For *D. luteipes* and *C. externa*, the prey were *S. frugiperda* egg masses containing 100 eggs, whereas for *E. connexa*, 50 eggs were used as prey. The barometric chamber was situated in an environmentally controlled room at 25 ± 1°C, 60 ± 10% RH, and a L:D 12:12 photoperiod.

**FIGURE 3:**
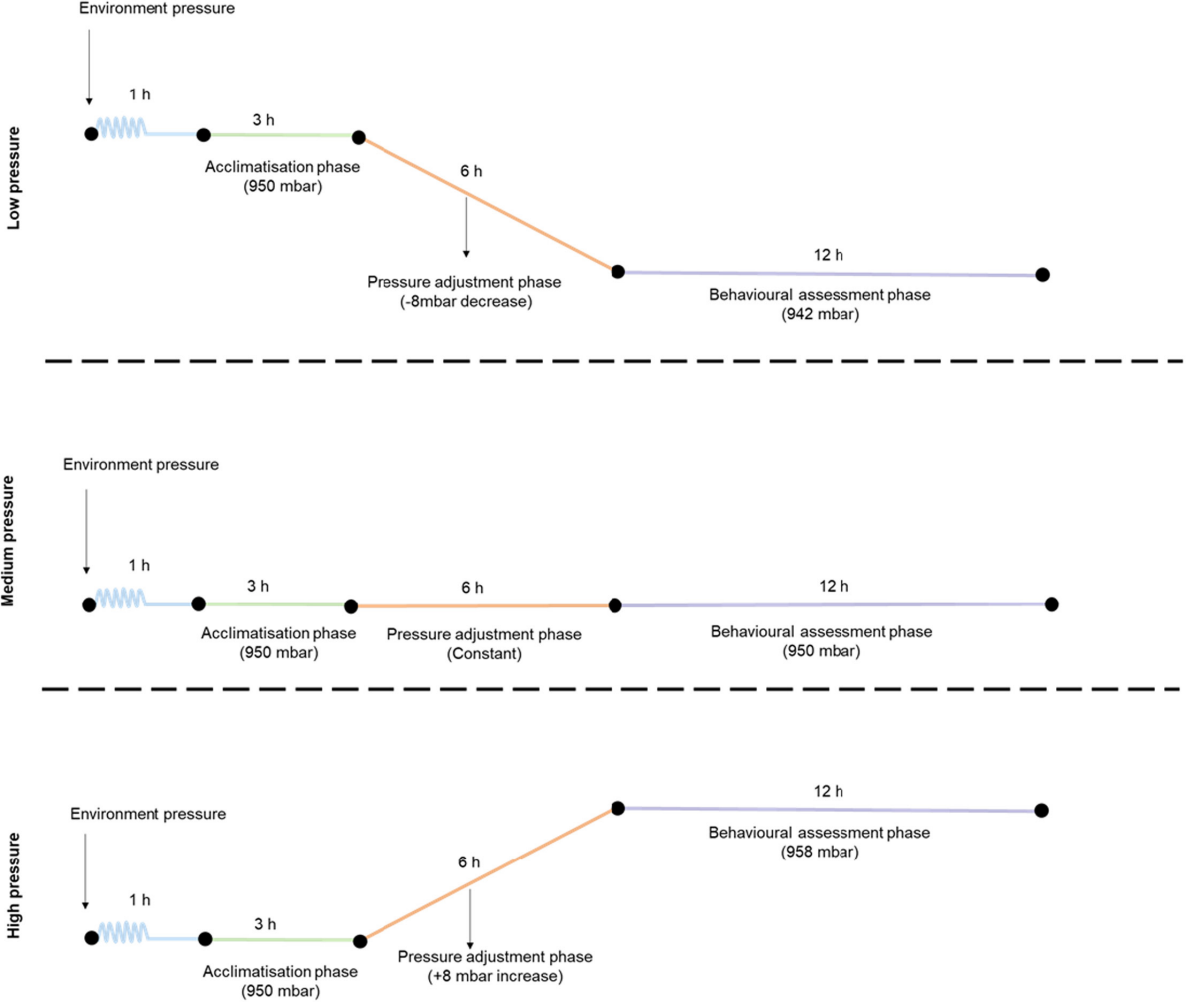
Barometric pressure ramps used to investigate the influence of pressure on the foraging activity of *Doru luteipes*, *Eriopis connexa* and *Chrysoperla externa*. The experiment began with an acclimatisation phase where the pressure was programmed to reach 950 ± 1 mbar within 1 h, followed by a 3‐h stabilization period. Subsequently, the pressure adjustment phase varied for each treatment. In the low‐pressure treatment, the pressure gradually decreased by 8 mbar, reaching a final pressure of 942 ± 1 mbar. In the medium‐pressure treatment, the pressure remained constant at 950 ± 1 mbar. In the high‐pressure treatment, the pressure gradually increased by 8 mbar until reaching a final pressure of 958 ± 1 mbar. The adjustment phase lasted for 6 h. Once the desired pressure regimes were achieved, they were maintained at a constant level for 12 h during the behavioural assessment phase.

After arranging the predators and prey inside the barometric chamber, it was sealed. The experiments followed specific pressure ramps based on the treatments (Figure 3). As the environment pressure naturally fluctuates, the pressure ramp was programmed to reach 950 ± 1 mbar within 1 h. This target pressure was then held for 3 h (acclimatisation phase). Following the acclimatisation phase, the pressure adjustment phase began and lasted 6 h. In the low‐pressure treatment, the pressure was gradually reduced by 8 mbar reaching a final pressure of 942 ± 1 mbar. In the medium‐pressure treatment, the pressure was maintained at a constant level of 950 ± 1 mbar. For high‐pressure treatment, the pressure was gradually increased by 8 mbar until reaching a final pressure of 958 ± 1 mbar. Once the desired pressure regimes were achieved, they were maintained at a constant level for a 12‐h period (behavioural assessment phase). During this phase, the predators were released to interact with the prey. All bioassays were recorded using a Sony HDR‐SR12. The foraging behaviour of *E. connexa* and *C. externa* was recorded during the photophase, while *D. luteipes* behaviour was recorded during the scotophase.

We assessed the searching time, the feeding time and the egg consumption rate according to Fonseca et al. (2000). The searching time (min) represents the duration it took for the predator to encounter the prey. Thus, it started upon releasing the predators from the upper compartment and persisted until they successfully encountered the prey. The feeding time (min) started when the predator made initial contact with the prey, encompassing both prey subjugation and consumption, and finished when the predator ceased feeding and departed from the egg masses. Occasionally, some predators returned to feed on the prey, but this additional feeding time was not considered in the calculation of the overall feeding time. The egg consumption rate (%) was determined by counting the number of eggs consumed by the predator within a 12‐h period. Eight replicates with five subsamples per treatment were conducted.

### 2.6 Statistical analyses

The searching time and feeding time were analysed using mixed effects Cox proportional hazards models, since they are times until an event. We used the package ‘coxme’ (Therneau, 2022) to fit these models. The egg consumption rate was analysed using a binomial generalized linear mixed model since it consists of discrete proportions. We used the package ‘lme4’ (Bates et al., 2015) to fit these models. The effect of life stage (immature vs. adult), barometric pressure (low, medium and high) and the two‐way interaction between life stage and barometric pressure were included as fixed in the linear predictor, while the effects of replicate were included as random (since subsamples within the same replicate are correlated). The significance of the effects was assessed using likelihood‐ratio tests for nested submodels. Goodness‐of‐fit was assessed using half‐normal plots with a simulated envelope, using package ‘hnp’ (Moral et al., 2017). This technique is used to assess whether the observed data are a plausible realization of a fitted model. All analyses were performed in R version 3.02.3 (R Core Team, 2020).

## 3 RESULTS

### 3.1 Impact of barometric pressure on *Doru luteipes* foraging behaviour

**FIGURE 4:**
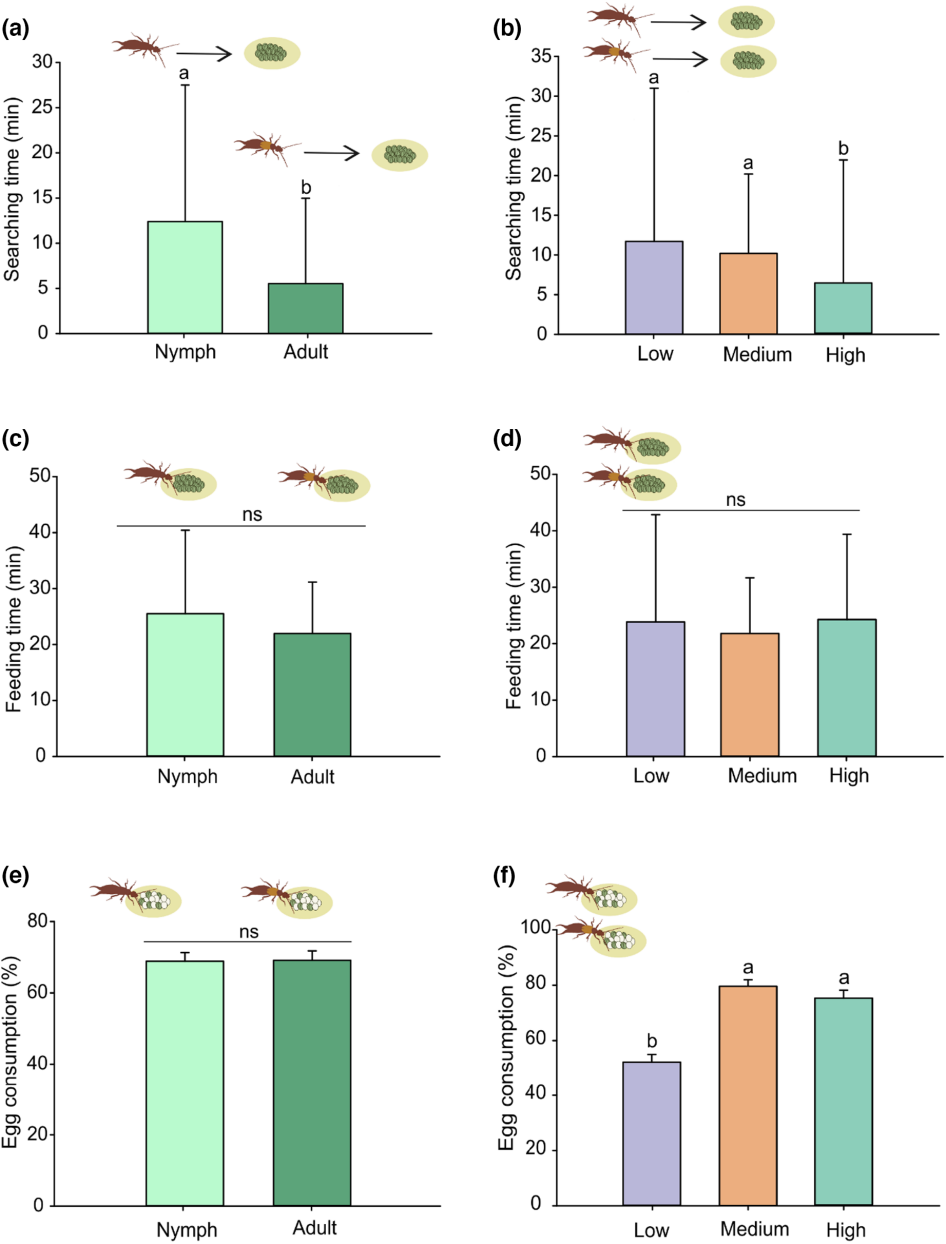
Marginal statistics for the foraging responses of *Doru luteipes* nymphs and adults to low, medium and high barometric pressure regimes. (a) Searching time (median ± SE in min) per life stage; (b) searching time (median ± SE in min) as a function of barometric pressure regime; (c) feeding time (median ± SE in min) per life stage; (d) feeding time (median ± SE in min) per barometric pressure regime; (e) consumption rate (mean ± SE) of *Spodoptera frugiperda* eggs per life stage; and (f) consumption rate (mean ± SE) of *S. frugiperda* eggs per barometric pressure regime. Bars with the same letters are not significantly different (Tukey–Kramer test, *p* < .05) (*n* = 8).

The searching time was significantly affected by the life stages of earwigs (GLMM: χ^2^ = 9.54; df = 1; *p* = .002) and barometric pressure regimes (GLMM: χ^2^ = 6.836; df = 2; *p* = .032), but there was no significant interaction between them (GLMM: χ^2^ = 0.52; df = 2; *p* = .769) (Table S1). Therefore, we only compared marginal means in this case (since the interaction was not significant). Nymphs spent significantly more time searching for prey than adults (Figure 4a). The searching time was significantly lower for both nymphs and adults under high barometric pressure compared to medium‐ and low‐pressure regimes (Figure 4b). Prey feeding time, on the contrary, was not affected by life stages (GLMM: χ^2^ = 2.496; df = 1; *p* = .287), barometric pressure regimes (GLMM: χ^2^ = 5.028; df = 2; *p* = .080) and their interaction (GLMM: χ^2^ = 2.496; df = 2; *p* = .287) (Figure 4c,d, Table S1). The egg consumption rate was significantly affected by barometric pressure regimes (GLMM: LR = 27.847; df = 2; *p* < .001), but was not affected by life stages (GLMM: LR = 0.446; df = 1; *p* = .503) nor its interaction with barometric pressure (GLMM: LR = 0.182; df = 2; *p* = .912). Both nymphs and adults consumed significantly fewer eggs under low barometric pressure compared with medium‐ and high‐pressure regimes (Figure 4f).

### 3.2 Impact of barometric pressure on *Eriopis connexa* foraging behaviour

**FIGURE 5:**
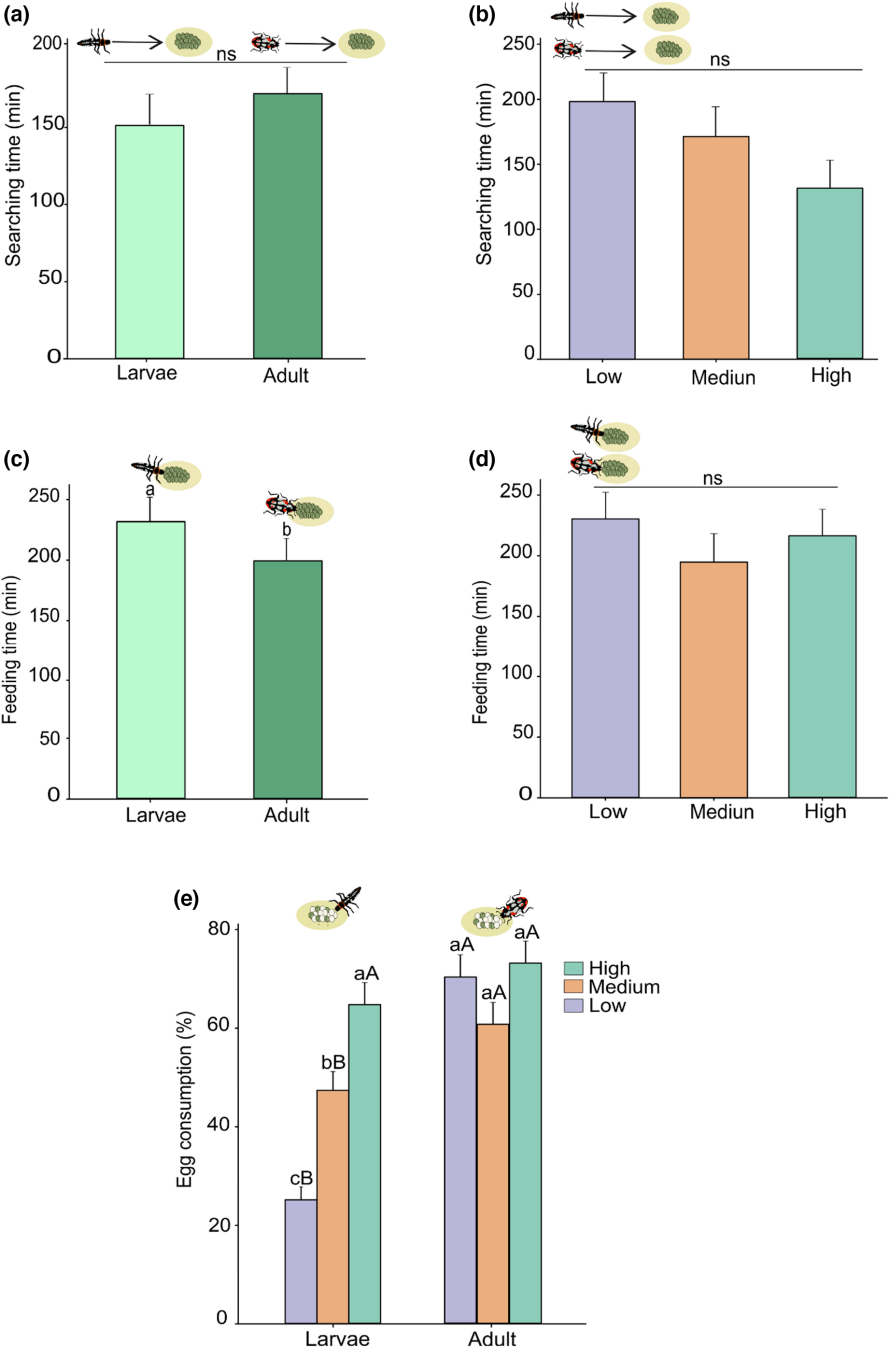
Marginal statistics for the foraging responses of *Eriopis connexa* larvae and adults to low, medium and high barometric pressure regimes. (a) Searching time (median ± SE in min) per life stage; (b) searching time (median ± SE in min) per barometric pressure regime; (c) feeding time (median ± SE in min) per life stage; (d) feeding time (median ± SE in min) per barometric pressure regime; and (e) consumption rate (mean ± SE) of *Spodoptera frugiperda* eggs per life stage and barometric pressure regime. Bars with the same letters are not significantly different (Tukey–Kramer test, *p* < .05) (*n* = 8). Lower‐case letters compare pressure regimes within the phase and upper‐case letters compare phases within pressure regimes.

Ladybird beetle searching time was not affected by life stages (GLMM: χ^2^ = 0.60; df = 1; *p* = .43), barometric pressure regimes (GLMM: χ^2^ = 5.57; df = 2; *p* = .06) and their interaction (GLMM: χ^2^ = 0.674; df = 2; *p* = .713) (Figure 5a,b, Table S1). The feeding time, however, was significantly affected by life stages (GLMM: χ^2^ = 5.23; df = 1; *p* = .02) in which larvae spent more time manipulating prey than adults (Figure 5c). Barometric pressure (GLMM: χ^2^ = 1.159; df = 2; *p* = .560) and its interaction with life stages (GLMM: χ^2^ = 5.519; df = 2; *p* = .063) did not affect the feeding time (Table S1). The egg consumption rate was significantly affected by the interaction of life stages and barometric pressure regimes (GLMM: LR = 18.12; df = 2; *p* < .001). Larvae egg consumption rate was significantly affected by barometric pressure regimes (GLMM: LR = 18.12; df = 2; *p* < .001) (Table S1). The higher consumption rate was registered at a high‐pressure regime, whereas the lower consumption was at low barometric pressure (Figure 5e). Adults, however, were not affected by barometric pressure regimes (Figure 5e).

### 3.3 Impact of barometric pressure on *Chrysoperla externa* foraging behaviour

**FIGURE 6:**
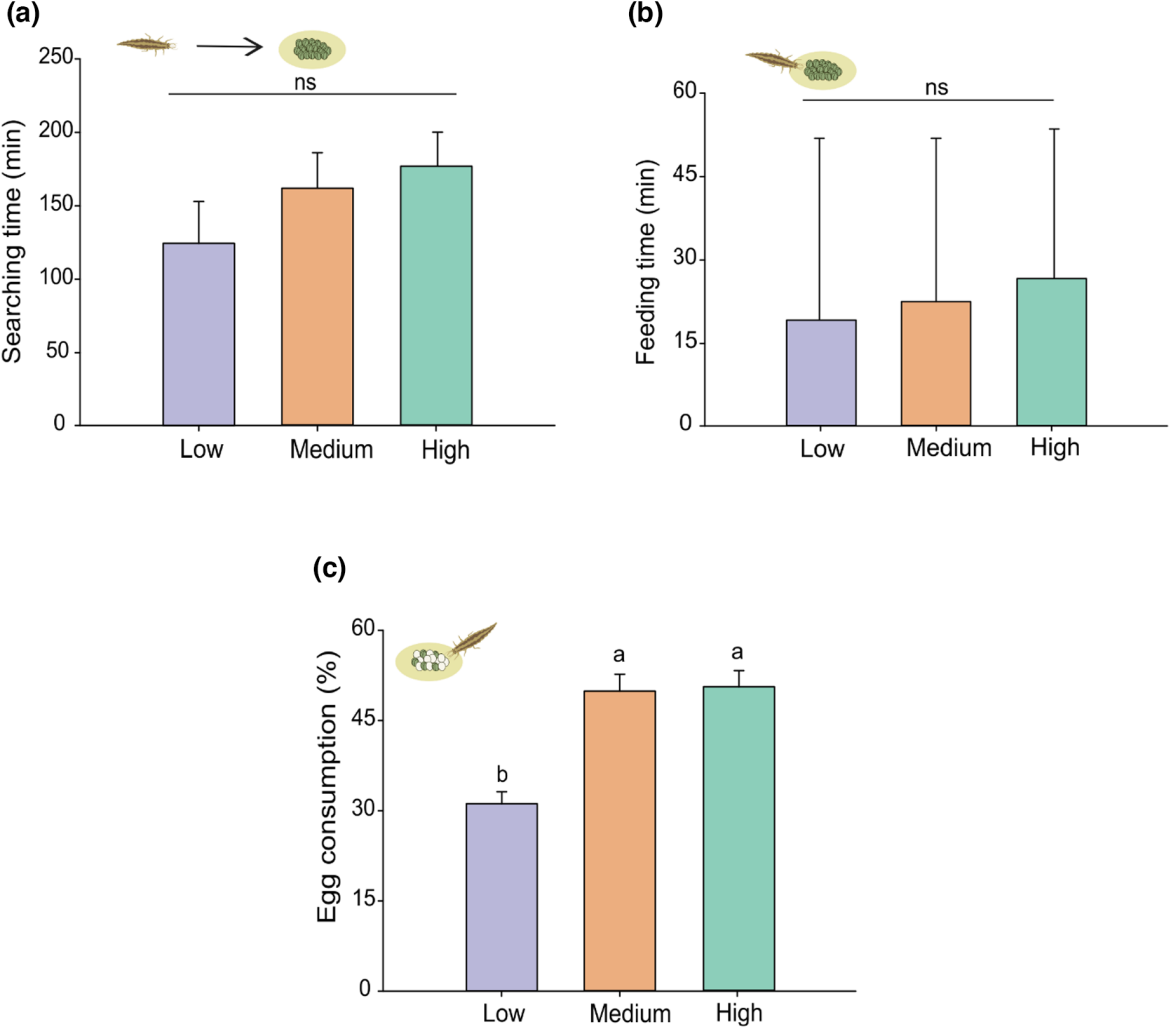
Marginal statistics for the foraging responses of *Chrysoperla externa* larvae to low, medium and high barometric pressure regimes. (a) Searching time (median ± SE in min) per barometric pressure regime; (b) feeding time (median ± SE in min) per barometric pressure regime; and (c) consumption rate (mean ± SE) of *Spodoptera frugiperda* eggs per barometric pressure regime. Bars with the same letters are not significantly different (Tukey–Kramer test, *p*‐value < .05) (*n* = 8).

Green lacewings searching time (GLMM: χ^2^ = 5.08; df = 2; *p* = .078) and feeding time (GLMM: χ^2^ = 2.49; df = 2; *p* = .287) were not affected by barometric pressure regimes (Figure 6a,b, Table S1). The egg consumption rate, however, was significantly affected by barometric pressure (GLMM: LR = 29.55; df = 2; *p* < .001). The egg consumption rate decreased at low‐pressure compared with medium‐ and high‐pressure regimes (Figure 6c).

## 4 DISCUSSION

Insects can perceive changes in environmental factors and modify their behaviour accordingly. These changes can include shifts in searching tactics, resource preferences or overall foraging strategies. Such adaptability is crucial for their survival in a dynamic environment (Hassell & Southwood, 1978). In this study, we have demonstrated that barometric pressure significantly impacts the foraging behaviour and consumption rate of entomophagous insects, with varying effects depending on species and life stages.

We showed that the earwig *D. luteipes* modulates its foraging behaviour in response to changes in barometric pressure. When exposed to a high‐pressure regime, earwigs initiated their search for prey more rapidly compared with when exposed to medium‐ or low‐pressure regimes. Notably, we found that changes in barometric pressure did not affect the duration of feeding time. However, under low‐pressure conditions, the consumption rate of earwigs was significantly reduced. Earwigs are characterized by their cryptic nature and thigmotropic behaviour, exhibiting a preference for maximum contact with the substrate's surface (Cruz, 2009; Jarvis et al., 2005). They are commonly found in maize and sugarcane whorls, as well as plant bracts, which provide protection against environmental stress (Fenoglio & Trumper, 2014; Naranjo‐Guevara et al., 2017). Although our experimental setup did not include shelters for the earwigs, our findings suggest that barometric pressure serves as a relevant cue for them to leave their sheltered locations and actively search for food. Low barometric pressure seems to be used as an indication of weather instability for earwigs, leading to a reduction in the consumption rate as a pre‐emptive response to potential risk conditions. Furthermore, precipitation has been observed to influence their foraging behaviour, resulting in reduced activity (Fenoglio & Trumper, 2014). Previous studies have shown that during rainy periods, the predation‐induced mortality of *S. frugiperda* and *Diatraea saccharalis* (Fabr.) eggs decreases (Fenoglio & Trumper, 2014; Varella et al., 2015). Our results indicated that under high and medium barometric pressure conditions, earwigs consumed significantly more eggs than low‐pressure conditions. Interestingly, after rainfall, the populations of pest crop insects tend to increase due to the limitation of natural enemy activity (Catling & Islam, 1995; Weisser et al., 1997). Rain is a significant mortality factor that affects not only prey availability but also impacts foraging behaviour (Kasper et al., 2008; Santos et al., 2020). As a result, when confronted with low‐pressure conditions, which are associated with strong winds and rain, predators might reduce their foraging efforts, resulting in lower consumption rates. These environmental cues enable foragers to adjust their behaviour and enhance their chances in natural settings (Fink & Völkl, 1995; Pascalis et al., 2022). Insects can reduce their mortality risk by minimizing the time spent feeding in adverse conditions, which can affect their consumption rate.

In contrast to earwigs, the duration of the searching time and feeding time of the ladybird beetle *E. connexa* was not affected by fluctuations in barometric pressure. Ladybird beetles exhibit different behaviour and habitat preferences compared with earwigs. They do not rely on shelters and instead spend a significant amount of time exploring the surrounding area in search of prey (Evans & Dixon, 1986). The duration of prey capture and subjugation by the ladybird beetle did not extend for long periods. The maximum feeding time observed was 230 ± 20.8 min under low‐pressure conditions, representing only one‐third of the total experimental time (720 min) (Figure 5d). In natural environments, drops in barometric pressure often precede the onset of strong winds and rain, which can persist for hours or even days, depending on various factors such as their intensity, atmospheric system and geographic location (Lazaridis, 2011). The time window between the pressure drop and the arrival of unstable weather may provide predators with an opportunity to capture prey and obtain enough energy for survival before unsettled conditions ensue. This strategy enables predators to prepare for potential food scarcity resulting from unstable weather conditions (Heupel et al., 2003; Matley et al., 2019; Sujimoto et al., 2019). Although changes in barometric pressure did not significantly affect their foraging behaviour, ladybird beetles consumed fewer eggs under low‐pressure conditions. It is noteworthy that the prey used in our study were *S. frugiperda* eggs, which have a thin and flexible tegument, lack mobility, and are unable to actively evade predators. These characteristics may have allowed both earwigs and ladybird beetles to maximize their prey feeding time under variations in barometric pressure.

The foraging behaviour (i.e. searching and feeding times) of the green lacewing *C. externa* remained unaffected by changes in barometric pressure. Similar to ladybird beetles, green lacewings are not reliant on shelters and possess a high locomotor capacity, rendering them more exposed to environmental factors (Fleschner, 1950; Freitas, 2001). Other abiotic factors, such as precipitation, do not appear to have a negative effect on the foraging behaviour of several lacewing species (Moelleman et al., 2015; Ramzan et al., 2019). Therefore, it is evident that different predatory insect species exhibit variations in their ability to explore habitats and respond to environmental changes (Comont et al., 2012, 2014). Interestingly, similar to the findings observed for earwigs and ladybird beetles, the consumption rate of the green lacewings was negatively influenced by low‐pressure conditions, resulting in a reduced number of preyed eggs compared with medium‐ and high‐pressure regimes. Other studies have shown that low temperatures and high CO_2_ levels also have a detrimental effect on the consumption rate of lacewings (Gao et al., 2010; Islam & Chapman, 2001; Kumar et al., 2011). Understanding the consumption rate of predators is crucial for evaluating their efficiency in biological control strategies aimed at managing insect pest populations (Solomon, 1949).

The ability of insects to adapt to biotic and abiotic factors varies depending on their developmental stage. We investigated this phenomenon by comparing the responses of *D. luteipes* and *E. connexa* immatures and adults to changes in barometric pressure. We found that searching and feeding times did not differ between life stages across the range of tested barometric pressure conditions. However, the ladybird beetle larvae consumed fewer eggs of *S. frugiperda* at a low‐pressure regime, while the adults' consumption rate was unaffected by barometric pressure. This might be likely due to the differences in the composition and structure of the tegument between these two life stages in holometabolous insects (Noh et al., 2016, 2017). Specifically, beetle larvae have soft and flexible cuticles, while adults have rigid and hard cuticles with resistant wings that protect them from environmental factors (Chen et al., 2017; Fraenkel & Rudall, 1940; Xing & Yang, 2020). Other studies have also shown that insects exhibit different adaptive responses to abiotic factors, such as temperature and humidity, depending on their life stage (Chen et al., 2017; Hennessy et al., 2015; Kingsolver et al., 2011; Nervo et al., 2021; Zhang et al., 2015). For example, *Colias* butterfly larvae use physiological mechanisms to cope with thermal variability (Sherman & Watt, 1973), while adults rely on morphological adaptations such as wing melanin and thickness of ventral thoracic bristles (Ellers & Boggs, 2004; Watt, 1969). Similarly, experiments on *Tribolium castaneum* (H.) adults revealed that individuals with intact elytra exhibited better protection against environmental stressors than those whose hind wings were mutilated (Linz et al., 2016). In contrast, despite variations in barometric pressure, immature and adult *D. luteipes* exhibit similar foraging behaviour. Earwigs, which are hemimetabolous insects, typically do not undergo drastic variations in morphological and ecological characteristics throughout life (Cruz, 2009; Reis et al., 1988; Truman, 2019). In maize fields, earwigs at all stages utilize the same strategy to shelter and protect themselves from environmental stressors such as desiccation, high temperatures, rain and solar radiation (Cruz, 2007; Sauphanor & Sureau, 1993). Nymphs and adult *Labidura riparia* (Pallas) earwigs show similarities in terms of heat tolerance and cuticular permeability (Kharboutli & Mack, 1993). This shared characteristic can be attributed to their brachyelytra wings. While adult earwigs possess more sclerotized anterior wings, these wings are shortened, leaving the abdomen exposed similar to nymphs. Consequently, both immature and adult earwigs may respond similarly to physical damage and stress (Haas & Kukalova‐Peck, 2001). These findings indicate that the structural characteristics of earwigs play a crucial role in their ability to withstand abiotic factors, thereby explaining the similar behaviour observed in both nymphs and adults during our experiments.

Moreover, insects face a trade‐off between allocating time and energy to feeding and avoiding both biotic and abiotic risks (Ferrari et al., 2009; Lima & Bednekoff, 1999). This trade‐off arises from the dilemma insects encounter when they must balance their resource needs with the imperative of protecting themselves from unfavourable conditions. Our results indicate that when risk is high (low barometric pressure), all three predators reduced their consumption rates while foraging duration (i.e. searching and feeding times) remained unaffected. It is possible that they optimized their foraging behaviour as a risk management strategy, resulting in a reduced consumption rate. Therefore, predatory insects are capable of detecting a decrease in barometric pressure, correlating it with environmental effects, and then adjusting their foraging behaviour accordingly. However, the increase in barometric pressure did not alter predators' behaviour and consumption rate compared with the medium‐pressure regime. It remains unclear whether they can detect pressure increases or whether they are not stimulated to change their foraging behaviour in response to this increase. Certain insect species, recognizing that the risk of mortality can have dire consequences for their survival and that of their offspring (Weisser et al., 1997), may prioritize seeking shelter and temporarily suspend foraging behaviour when unsettled weather conditions are imminent. For instance, parasitoids such as *Encarsia formosa* (Van Roermund & Van Lenteren, 1995), *Aphidius nigripes* (Marchand & McNeil, 2000) and *Cotesia glomerata* (Steinberg et al., 1992) reduce their foraging activities in weather conditions associated with rain, strong winds and a decline in barometric pressure. On the contrary, species such as *Apis mellifera* exhibit increased foraging activity one day before rainfall to secure reserves in case adverse weather disrupts their food sources (He et al., 2016). Similarly, the leaf‐cutter ants *Atta sexdens* enhance their foraging efficiency by cutting and transporting a larger number of leaves to their nests when the barometric pressure drops (Sujimoto et al., 2019). These changes in foraging behaviour, prompted by a decrease in barometric pressure, demonstrate that insects are capable of aligning their needs and abilities when making decisions in the face of risks. While researchers have explored how insects perceive variations in brightness (Van Der Kooi et al., 2021), odours (Montagné et al., 2015), temperature, and humidity (Nurme et al., 2015), the morphological structures responsible for perceiving barometric pressure in insects remain unknown (McIver, 1984; Tichy & Kallina, 2010).

In summary, our study showed that three predatory insects from different taxonomic groups, with varying morphological, physiological, ecological and biological characteristics, modify their foraging behaviour in response to barometric pressure conditions. Notably, earwigs responded more quickly to favourable high‐pressure regimes in the initial stages of foraging (searching time), whereas the green lacewings and the ladybird beetle had similar searching times under varying pressure regimes. While there were no differences in prey feeding time among the studied species at any pressure regimes, the consumption rate was found to be influenced by barometric pressure. Specifically, low‐pressure conditions had a negative impact on consumption rates. Moreover, our study provides novel insights into the response of hemimetabolous (*D. luteipes*) and holometabolous (*E. connexa*) species at different life stages to barometric pressure. We demonstrated for the first time that insects from these two types of metamorphosis exhibit distinct responses to changes in barometric pressure: earwig nymphs and adults displayed similar consumption rates across all pressure regimes tested, whereas the ladybird beetle larvae consumed fewer eggs under low‐pressure conditions, but adults were unaffected. However, since we only included one species to represent hemimetabolous and holometabolous groups, future studies should incorporate a more diverse range of species to strengthen the conclusions drawn from our study. Overall, our findings have direct implications for sustainable agriculture, as these predator insects are important biological control agents and the observed effects of barometric pressure on their foraging behaviour could have practical implications for their performance in the field. The challenge now is to understand which structures in insects are responsible for detecting barometric pressure variations.

## AUTHOR CONTRIBUTIONS

**Kamila E. X. Azevedo:** Conceptualization (equal); data curation (equal); formal analysis (equal); investigation (lead); methodology (lead); project administration (equal); resources (equal); writing – original draft (equal); writing – review and editing (equal). **Diego M. Magalhaes:** Investigation (equal); supervision (equal); writing – original draft (equal); writing – review and editing (equal). **Rafael de Andrade Moral:** Formal analysis (lead); methodology (supporting). **José Maurício S. Bento:** Conceptualization (equal); funding acquisition (lead); investigation (equal); resources (lead); supervision (lead); writing – review and editing (equal).

## FUNDING INFORMATION

This research was financially supported by the National Institute of Science and Technology of Semiochemicals in Agriculture (INCT), Fundação de Amparo à Pesquisa do Estado de São Paulo (FAPESP) and Conselho Nacional de Desenvolvimento Científico (CNPq) [grants 2014/50871‐0 and 465511/2014‐7 to JMSB; 2019/24492‐6 to DMM]. KEXA was supported by a doctoral scholarship from Coordenação de Aperfeiçoamento de Pessoal de Nível Superior (CAPES, Process: 88887.469398/2019‐00) and CNPq (Process: 140268/2020‐0) Brazil.

## CONFLICT OF INTEREST STATEMENT

We have no conflicts of interest to disclose.

## Supporting information


Table S1.

## Data Availability

Data deposited in the Figshare digital repository: https://doi.org/10.6084/m9.figshare.22564174. The DOI becomes active when the item is published, but you can access the data through this private link: https://figshare.com/s/3c2bf312911b520fc318.
