# Supplementary material for: Weathering the hunt: The role of barometric pressure in predator insects' foraging behaviour
Source: Ecol Evol. 2023 Aug 9;13(8):e10416. doi: 10.1002/ece3.10416 (PMC10412439; doi:10.1002/ece3.10416)
Supplement: Supplementary file 1 — Table S1. [file ECE3-13-e10416-s001.docx]

**Supporting information**

**Table 1.** Statistical tests and associated degrees of freedom (d.f.) and p-values for the effects included in the linear predictors for the models fitted to the three species' data.

| ***Doru luteipes*** |  |  |  |  |  |
| --- | --- | --- | --- | --- | --- |
| **Variable** | **Effect** | **Statistical test** | **d.f.** | **p-value** | **Estimated random effect variance** |
|  | Life stage | 3,94 | 1 | 0,047 |  |
| Feeding time | Pressure | 5,03 | 2 | 0,081 | 0,0004 |
|  | Interaction | 0,27 | 2 | 0,875 |  |
|  | Life stage | 9,54 | 1 | 0,002 |  |
| Searching time | Pressure | 6,84 | 2 | 0,033 | 0,031 |
|  | Interaction | 0,52 | 2 | 0,770 |  |
|  | Life stage | 0,45 | 1 | 0,504 |  |
| Egg consumption | Pressure | 27,85 | 2 | < 0.001 | 0,680 |
|  | Interaction | 0,18 | 2 | 0,913 |  |
|  |  |  |  |  |  |
| ***Chrysoperla externa*** | |  |  |  |  |
| **Variable** | **Effect** | **Statistical test** | **d.f.** | **p-value** | **Estimated random effect variance** |
| Feeding time | Pressure | 2,50 | 2 | 0,287 | 0,00008 |
| Searching time | Pressure | 5,09 | 2 | 0,079 | 0,0004 |
| Egg consumption | Pressure | 29,56 | 2 | < 0.001 | 0,079 |
|  |  |  |  |  |  |
| ***Eriopis connexa*** |  |  |  |  |  |
| **Variable** | **Effect** | **Statistical test** | **d.f.** | **p-value** | **Estimated random effect variance** |
|  | Life stage | 5,24 | 1 | 0,022 |  |
| Feeding time | Pressure | 1,16 | 2 | 0,560 | 0,00008 |
|  | Interaction | 5,52 | 2 | 0,063 |  |
|  | Life stage | 0,60 | 1 | 0,438 |  |
| Searching time | Pressure | 5,57 | 2 | 0,062 | 0,00008 |
|  | Interaction | 0,67 | 2 | 0,714 |  |
|  | Life stage | 24,68 | 1 | < 0.001 |  |
| Egg consumption | Pressure | 15,73 | 2 | < 0.001 | 0,375 |
|  | Interaction | 18,21 | 2 | < 0.001 |  |
